# Malaria among under-five children in rural communities of Al-Mahweet governorate, Yemen

**DOI:** 10.1186/s12936-022-04371-8

**Published:** 2022-11-18

**Authors:** Mona A. A. Al-Quhaiti, Rashad Abdul-Ghani, Mohammed A. K. Mahdy, Methaq A. Assada

**Affiliations:** 1 Laboratory Department, Ibn Sina Specialist Hospital, Sana’a, Yemen; 2 Department of Medical Parasitology, Faculty of Medicine and Health Sciences, Sana’a University, Sana’a, Yemen; 3 Tropical Disease Research Center, Faculty of Medicine and Health Sciences, University of Science and Technology, Sana’a, Yemen; 4 National Malaria Control Programme, Ministry of Public Health and Population, Sana’a, Yemen

**Keywords:** Falciparum malaria, Under-five children, Prevalence, Risk factors, Yemen

## Abstract

**Background:**

Malaria burden among under-five children living in endemic areas of Yemen is largely unknown due to the lack of community-based studies. Therefore, this study determined the prevalence and risk factors associated with falciparum malaria among under-five children in rural communities of Al-Mahweet governorate, Yemen.

**Methods:**

This community-based, cross-sectional study recruited 400 under-five children from two rural districts of Al-Mahweet governorate in December 2019. Demographic characteristics (gender, age, education and occupation of the child’s parents, and household size) and risk factors associated with malaria were collected through interviews with children’s caregivers using a structured questionnaire. Finger-prick blood was screened for *Plasmodium falciparum* and non-falciparum species using rapid diagnostic tests (RDTs), and duplicate Giemsa-stained thick and thin blood films were examined for malaria parasites. The density of asexual *P. falciparum* stages was also estimated. Data were then analysed, and the agreement between the results of thick-film microscopy and RDTs for diagnosing falciparum malaria was assessed using the kappa index. Statistical significance was set at a *P*-value of < 0.05.

**Results:**

*Plasmodium falciparum* was prevalent among 9.8% (95% CI 7.0–13.1) of under-five children in the rural communities of Al-Mahweet, with a median asexual parasite density of 763 ± 2606 parasites/μl of blood (range: 132–4280) and low-to-moderate parasitaemia levels. Approximately one-third of microscopy-confirmed cases were gametocyte carriers. Multivariable logistic regression analysis confirmed that age of three years or older (AOR = 5.6, 95% CI 1.6–19.8; *P* = 0.007), not sleeping under a mosquito net the previous night of the survey (AOR = 8.0, 95% CI 2.4–27.4; *P* = 0.001), sleeping outdoors at night (AOR = 4.4, 95% CI 2.0–10.0; *P* < 0.001), and absence of indoor residual spraying (IRS) during the last year (AOR = 4.2, 95% CI 1.9–9.4; *P* < 0.001) were the independent predictors of falciparum malaria among under-five children in the rural communities of Al-Mahweet. The observed percentage agreement between thick-film microscopy and RDTs was 98.5%, with a very good agreement (*k-*index = 0.9) between the two methods for falciparum malaria diagnosis that was statistically significant.

**Conclusion:**

Approximately one in ten under-five children in rural communities of Al-Mahweet is infected with *P. falciparum* based on microscopy and RDTs. Age of three years or older, not sleeping under mosquito nets, sleeping outdoors at night and absence of IRS can independently predict falciparum malaria among them. The very good agreement between thick-film microscopy and RDTs for diagnosing falciparum malaria in children supports the usefulness of using RDTs in such resource-limited rural communities.

## Background

Malaria is a major public health problem in the world. According to the latest report by the World Health Organization (WHO), 241 million cases were estimated in 85 malaria-endemic countries, increasing from 227 million in 2019 [1]. In the WHO Eastern Mediterranean Region, 5.7 million cases and 12,330 deaths were estimated in 2020 [1]. Despite the efforts devoted by the National Malaria Control Programme (NMCP) since the early 2000s, the at-risk population was estimated at 64.5% and the reported cases exceeded 164,000 in 2020, with approximately 99% being caused by *Plasmodium falciparum* [1].

Under-five children are at high risk of contracting malaria and developing severe disease [2]. Apart from symptomatic cases, asymptomatic children can be major reservoirs of malaria, contributing substantially but silently to its transmission in endemic areas [3, 4]. Infection in asymptomatic carriers with microscopic parasitaemia often persists for months and goes undetected and untreated, making them a major gametocyte reservoir for mosquitoes [5, 6]. Children can be a key contributor to the infection of mosquitoes. For instance, a cohort study found that school-aged Ugandan children may be responsible for more than half of all mosquito infections [4]. Moreover, gametocytes from asymptomatic carriers can be more infectious to mosquitoes compared to those from symptomatic patients [7]. Unveiling the burden of asymptomatic malaria among children is instrumental in the success of malaria elimination interventions.

In Yemen, community-based malaria prevalence in under-five children is largely unknown, with few published studies reporting on childhood malaria. In this regard, 18.6% of children in rural districts of Taiz governorate were confirmed to be infected with *P. falciparum* in the mid-2000s, and falciparum malaria was recently reported among 8% of schoolchildren from Bajil district of Hodeidah governorate [8, 9]. Another study found that suspected severe falciparum malaria may account for up to 40% of paediatric admissions during the peak transmission season in endemic areas, and more than half of confirmed cases can be severe [10]. With the prevalence of malnutrition in endemic areas as a result of the humanitarian crisis imposed by armed conflicts [11], children are at high risk of malaria morbidity and mortality. Therefore, the present study determined the prevalence and risk factors associated with falciparum malaria among under-five children in the rural communities of Al-Mahweet during the peak transmission season. In addition, agreement between rapid diagnostic tests (RDTs) and thick-film microscopy for diagnosing falciparum malaria among rural children was assessed.

## Methods

### Study design, population and setting

A community-based, cross-sectional study was conducted among under-five children in two rural districts of Al-Mahweet governorate known to be highly endemic for malaria in December 2019. Children of both genders were included in the study if they were residents in the study districts over the year preceding the study and did not receive antimalarial drug(s) one month before the study, and if their parents/guardians gave written informed consent.

**Fig. 1:**
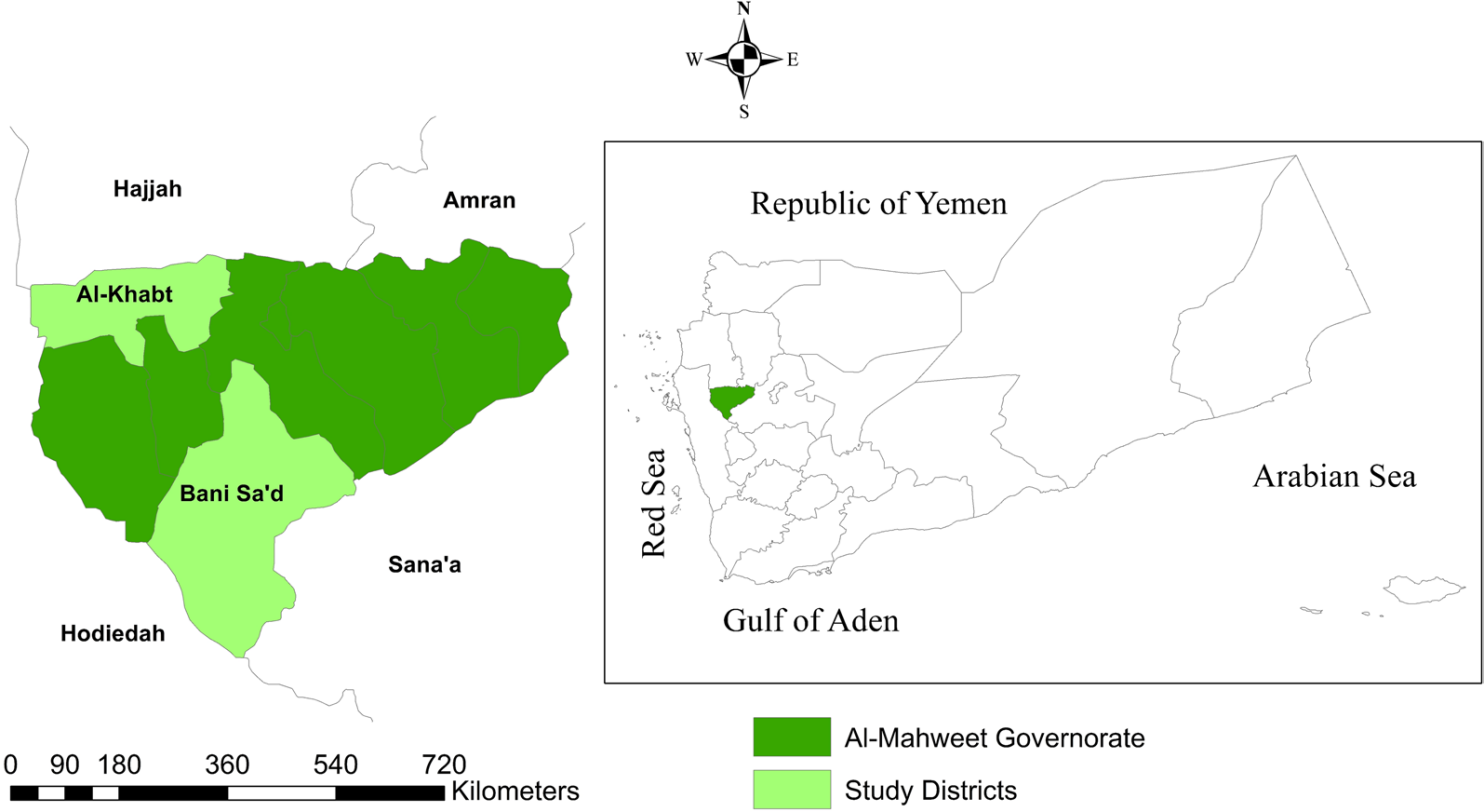
Map of Yemen showing the study governorate and districts

Al-Mahweet is a small mountainous governorate at the coordinates of 15° 28′ N and 43° 32′ E, approximately 111 km to the northwest of Sana'a. It is about 2,382 km^2^ and is inhabited by approximately 597,000 people [12]. It is endemic for malaria and borders two governorates with the highest burden of malaria in Yemen [13], Hodeidah to the west and Hajjah to the north. The epidemiology of malaria in Al-Mahweet might be influenced by internal displacement from its bordering highest-burden governorates as a result of the armed conflicts. The malaria transmission season in the governorate usually lasts for approximately six months, from November to April [14]. Of its nine districts, Bani Sa’d and Al-Khabt (Fig. 1) are the most afflicted with malaria because of their low altitude (below 1500 m). In 2019*, P. falciparum* was detected among 15.6% (3235/20764) of febrile patients examined by RDTs and/or microscopy at health facilities in the two districts; of whom, 908/5001 (18.2%) were under-five children according to the data derived from the Electronic Disease Early Warning System (eDEWS) of Yemen (Dr Reema Alyusfi, eDEWS Manager, personal communication). Therefore, these two districts were purposively selected to conduct this study.

### Sample size and sampling strategy

A minimum sample size of 348 children was calculated using OpenEpi software, Version 3.01 (www.OpenEpi.com) based on an expected prevalence of 13% [8, 9], a confidence level of 95%, an absolute precision of 5% and a design effect of 2. Based on an expected non-response rate of 15%, the sample was adjusted to 400 children to account for ineligible or missing children due to absenteeism or caregivers’ refusal. A multi-stage cluster sampling strategy was adopted. First, villages of Bani Sa’d and Al-Khabt districts were listed, and 20 villages were randomly selected from the two districts. Second, 20 households with at least one under-five child were randomly selected from each village, followed by random sampling of one child if the household had more than one.

### Data and blood collection

Demographic characteristics (gender, age, education and occupation of the child’s parents and household size) and risk factors associated with malaria were collected through face-to-face interviews with children’s caregivers using a structured questionnaire. Axillary temperature was measured using a thermometer. Children with a temperature of ≥ 37.5 °C were considered febrile. Blood drops were collected by finger prick for rapid malaria testing and preparing duplicate thick and thin blood films.

### Malaria rapid testing and microscopy

Blood drops were screened for *P. falciparum* and non-falciparum species using CareStart™ Malaria HRP2/pLDH (Pf/PAN) Combo RDTs (AccessBio, NJ, USA), according to the manufacturer’s instructions. Such RDT kits are currently adopted by the NMCP for malaria diagnosis in the country. Thick and thin blood films were prepared, Giemsa-stained and examined microscopically using standard procedures [15, 16]. Thick films were examined for a minimum of 100 oil-immersion fields before being recorded as negative for malaria parasites. Parasite density per microlitre (µl) of blood was estimated by counting *P. falciparum* asexual stages against 200 leukocytes on thick blood films, assuming an average leukocyte count of 8000/µl of blood [15, 16]. This procedure was repeated in two other areas of the film, and the average of the three counts was then recorded.

### Data analysis

Data were analysed using IBM SPSS Statistics, Version 23.0 (IBM Corp., Armonk, NY, USA). Categorical variables were expressed as frequencies and proportions, while continuous variables were expressed as the median and interquartile range (IQR) for non-normally distributed data. The prevalence of falciparum malaria was reported with its corresponding 95% confidence interval (CI), as determined by microscopy and/or RDTs. Univariate analysis with Pearson's chi-square or Fisher’s exact test was used to test the association of demographic and clinical characteristics as well as risk factors with falciparum malaria among under-five children, reporting the odds ratios (ORs) and corresponding 95% CIs of the associations. Multivariable logistic regression analysis was performed for significant factors in univariate analysis to identify the independent predictors of falciparum malaria among under-five children, together with their adjusted ORs (AORs) and corresponding 95% CIs. Agreement between the results of thick-film microscopy and RDTs for diagnosing falciparum malaria was assessed using the kappa (*k*) index, where the agreement was interpreted as poor (*k* < 0.20), fair (*k* = 0.20–0.40), moderate (*k* > 0.40–0.60), good (*k* > 0.60–0.80) or very good (*k* > 0.80) [17]. Statistical significance was set at a *P*-value of < 0.05.

## Results

### Characteristics of the study population

The majority of children included in this study were males (54.2%), aged three years or older (62.5%), with a median (IQR) age of 3 (2) years, and living in households of six members or more (63.5%). Most children’s fathers had primary education (37.0%) or were uneducated (37.0%) and were labourers (68.8%), while the majority of children’s mothers were uneducated (83.2%) and unemployed (99.2%) (Table 1).

**Table 1:** Characteristics of surveyed under-five children in rural communities of Al-Mahweet governorate, Yemen (2019)

Characteristics	*n*	(%)
Gender		
Male	217	(54.2)
Female	183	(45.8)
Age (years)		
< 3	150	(37.5)
≥ 3	250	(62.5)
Median (IQR): 3 (2)		
Household size (members)		
< 6	146	(36.5)
≥ 6	254	(63.5)
Father’s educational status		
Uneducated	148	(37.0)
Informal education	52	(13.0)
Primary education	148	(37.0)
Secondary education	38	(9.5)
University and above	14	(3.5)
Father’s occupation^*a*^		
Unemployed	103	(25.9)
Public service employee	19	(4.8)
Private sector employee	2	(0.5)
Labourer	274	(68.8)
Mother’s educational status		
Uneducated	333	(83.2)
Informal education	13	(3.3)
Primary education	50	(12.5)
Secondary education and above	4	(1.0)
Mother’s occupation		
Unemployed^b^	397	(99.2)
Public service employee	1	(0.3)
Private sector employee	2	(0.5)

The total number was 400

*IQR* interquartile range

^a^two missing cases

^b^mostly housewives

### Prevalence of falciparum malaria among under-five children

Falciparum malaria was prevalent among 9.8% (95% CI 7.0–13.1) of under-five children in rural communities of Al-Mahweet, where microscopy confirmed infection among 9.3% (95% CI 6.6–12.5) and RDTs were positive among 8.8% (95% CI 6.2–12.0) of children (Table 2).

**Table 2:** Prevalence of falciparum malaria among under-five children in rural communities of Al-Mahweet governorate, Yemen (2019)

Prevalence of falciparum malaria	*n*	(%)	95% CI
Microscopy-confirmed	37	(9.3)	(6.6–12.5)
RDT-positive	35	(8.8)	(6.2–12.0)
Total (microscopy and/or RDT)	39	(9.8)	(7.0–13.1)

The total number of children included in the study was 400

*CI* confidence interval

### Parasite density and gametocyte carriage

The median asexual parasite density among children with microscopy-confirmed falciparum malaria was 763 ± 2606 parasites/μl of blood (range: 132–4280), with low and moderate parasitaemia levels being prevalent among 51.4% and 48.6% of infected children, respectively. Of children with falciparum malaria, 32.2% were gametocyte carriers (Table 3).

**Table 3:** Parasite density and gametocyte carriage among *P. falciparum*-infected under-five children in rural communities of Al-Mahweet governorate, Yemen (2019)

Parasite indices	*n*	(%)
Parasite density (parasites/μl)		
Median ± IQR 763 ± 2606		
Range: 132–4280		
Parasitaemia levels		
Low (< 1000)	19	(51.4)
Moderate (1000–9999)	18	(48.6)
Gametocytaemia		
Yes	12	(32.4)
No	25	(67.6)

Calculated for microscopy-confirmed cases (37)

*IQR* interquartile range

### Association of children’s demographics and clinical characteristics with falciparum malaria

The prevalence of falciparum malaria was significantly higher among children aged three years or older (OR = 8.2, 95% CI 2.5–27.3; *P* < 0.001) than in younger children (14.4% *vs.* 2.0%, respectively). In contrast, no significant association was observed between falciparum malaria and gender, household size, parental educational status, or parental employment status. Likewise, no significant association was observed between falciparum malaria and fever, sweating, chills, vomiting, or jaundice (Table 4).

**Table 4:** Association of demographic and clinical characteristics with falciparum malaria among under-five children in Al-Mahweet governorate, Yemen (2019)

Characteristics	*N*	Falciparum malaria positivity				
		*n*	(%)	OR	(95% CI)	*P*-value
Gender						
Male	217	24	(11.1)	1.4	(0.7–2.7)	0.336
Female	183	15	(8.2)	Reference		
Age (years)						
≥ 3	250	36	(14.4)	8.2	(2.5–27.3)	< 0.001
< 3	150	3	(2.0)	Reference		
Household size (members)						
≥ 6	254	30	(11.8)	2.0	(0.9–4.4)	0.067
< 6	146	9	(6.2)	Reference		
Father’s educational status						
Literate	252	22	(8.7)	Reference		0.370
Illiterate	148	17	(11.5)	1.4	( 0.7–2.6)	
Mother’s educational status						
Literate	67	4	(6.0)	Reference		0.253
Illiterate	333	35	(10.5)	1.9	(0.6–5.4)	
Father’s employment status						
Employed	21	2	(0.5)	Reference		1.000
Unemployed	377	36	(9.0)	1.0	(0.2–4.5)	
Mother’s employment status						
Employed	3	0	(0.0)	NA		1.000
Unemployed	397	39	(9.8)			
Fever						
Yes	71	11	(15.5)	2.0	(0.9–4.2)	0.072
No	329	28	(8.5)	Reference		
Sweating						
Yes	28	5	(17.8)	2.2	(0.8–6.1)	0.134
No	372	34	(9.1)	Reference		
Chills						
Yes	7	1	(14.3)	1.5	(0.2–9.3)	0.515
No	393	38	(9.7)	Reference		
Vomiting						
Yes	45	2	(4.4)	0.4	(0.1–1.7)	0.205
No	355	37	(10.4)	Reference		
Jaundice						
Yes	4	1	(25.0)	3.1	(0.3–31.0)	0.301
No	396	38	(9.6)	Reference		

*N* number examined, *n* number positive, *OR* odds ratio, *CI* confidence interval, *NA* not applicable

### Risk factors associated with falciparum malaria among under-five children

Not sleeping under a mosquito net the previous night of the survey (OR = 11.8, 95% CI 3.6–39.0; *P* < 0.001), sleeping outdoors at night (OR = 4.5, 95% CI 2.2–9.2; *P* < 0.001), absence of indoor residual spraying (IRS) during the last year (OR = 3.4, 95% CI 1.7–6.8; *P* < 0.001) were significantly associated with falciparum malaria among under-five children in Al-Mahweet governorate (Table 5).

**Table 5:** Risk factors associated with falciparum malaria among under-five children in rural communities of Al-Mahweet governorate, Yemen (2019)

Factors	*N*	Falciparum malaria positivity				
		*n*	(%)	OR	(95% CI)	*P*-value
Sleeping under a mosquito net the previous night of the survey						
Yes	182	3	(1.6)	Reference		< 0.001
No	218	36	(16.5)	11.8	(3.6–39.0)	
Sleeping outdoors at night						
Yes	64	16	(25.0)	4.5	(2.2–9.2)	< 0.001
No	336	23	(6.8)	Reference		
IRS during the last year						
Yes	240	13	(5.4)	Reference		< 0.001
No	160	26	(16.3)	3.4	(1.7–6.8)	
Use of mosquito repellents						
Yes	0	0	(0.0)	NA		NA
No	400	39	(9.8)			
Residence in proximity to water collections						
Yes	298	32	(10.7)	1.6	(0.7–3.4)	0.255
No	102	7	(6.9)	Reference		
Residence in proximity to garbage collections						
Yes	187	19	(10.2)	1.1	(0.6–2.0)	0.795
No	213	20	(9.4)	Reference		
Screening windows						
Yes	55	1	(1.8)	Reference		0.064
No	345	38	(11.0)	6.7	(0.9–49.7)	

*N* number examined, *n* number positive, *OR* odds ratio, *CI* confidence interval, *IRS* indoor residual spraying, *NA* not applicable

### Independent predictors of falciparum malaria among under-five children

Multivariable logistic regression analysis confirmed that age of three years or older (AOR = 5.6, 95% CI 1.6–19.8; *P* = 0.007), not sleeping under a mosquito net the previous night of the survey (AOR = 8.0, 95% CI 2.4–27.4; *P* = 0.001), sleeping outdoors at night (AOR = 4.4, 95% CI 2.0–10.0; *P* < 0.001), and absence of IRS during the last year (AOR = 4.2, 95% CI 1.9–9.4; *P* < 0.001) were the independent predictors of falciparum malaria among under-five children in the rural communities of Al-Mahweet (Table 6).

**Table 6:** Independent predictors of falciparum malaria among under-five children in rural communities of Al-Mahweet governorate, Yemen (2019)

Independent predictors	AOR	(95% CI)	*P*-value
Age of three years or older	5.6	(1.6–19.8)	0.007
Not sleeping under a mosquito net the night before the survey	8.0	(2.4–27.4)	0.001
Sleeping outdoors at night	4.4	(2.0–10.0)	< 0.001
Absence of IRS during the last year	4.2	(1.9–9.4)	< 0.001

*AOR* adjusted odds ratio, *CI* confidence interval, *IRS* indoor residual spraying

### Agreement between thick-film microscopy and RDTs for malaria diagnosis

The results of thick-film microscopy and RDTs were concordant in all cases, except for two positive cases using RDTs and four positive cases by thick-film microscopy. The observed percentage agreement between the two methods was 98.5%, and there was very good agreement (*k* = 0.9) between thick-film microscopy and RDTs for falciparum malaria diagnosis that was statistically significant (Table 7).

**Table 7:** Agreement between thick-film microscopy and RDTs in detecting *P. falciparum* among under-five children in rural communities of Al-Mahweet governorate, Yemen (2019)

RDT result	Microscopy result		
	Negative	Positive	Total
Negative	361	4	365
Positive	2	33	35
Total	363	37	400

The observed agreement rate (361 + 33) × 100/400) = 98.5%; (*k* = 0.9; *P* < 0.001)

## Discussion

The vision of the NMCP’s strategic plan was to make Yemen malaria-free by the end of 2018 [18], but the disease remains a major public health problem in the country. Since 2015, the efforts to control and eliminate malaria have been hampered by the armed conflict and its repercussions, including the deteriorated health system, humanitarian crisis and internal displacement. This is the first study to report on the prevalence and risk factors associated with falciparum malaria in under-five children at the community level in Al-Mahweet governorate, Yemen. The prevalence of *P. falciparum* among 9.8% of under-five children in the rural communities of Al-Mahweet governorate is quite alarming, particularly with the asymptomatic nature of infection in the majority of surveyed children. Because this study has been conducted before the coronavirus disease 2019 (COVID-19) pandemic, the situation might be even worse as a result of the negative impact of the pandemic on the prevention and control of infectious diseases, including malaria. The prevalence of falciparum malaria among children in the present study is slightly higher than that (8%) recently reported among schoolchildren in Bajil district of Hodeidah [9], but lower than the microscopy-confirmed prevalence of 18.6% reported for *P. falciparum* among children in two rural districts of Taiz in the mid-2000s [8]. The absence of infection with *P. vivax* among under-five children in the present study is consistent with the very low prevalence of less than 0.25% recently reported among schoolchildren in Bajil [9].

The prevalence of falciparum malaria among under-five children in the present study is comparable to the microscopy-based prevalence of 11.7% reported among under-five children in southern highland Rwanda in community- and facility-based surveys [19]. Nonetheless, the polymerase chain reaction (PCR)-based prevalence among Rwandan children increased to 16.7% [19], highlighting the need for molecular surveillance of *P. falciparum* infection among children in the rural communities of Al-Mahweet for the true burden of infection to be uncovered. Compared to the present study, a higher prevalence was reported among under-five children in Tanzania (15.9% by RDTs) [20], Uganda (19.7% by microscopy) [21], sub-Saharan African countries (18.8–24.2% by microscopy and RDTs) [22], Nigeria (27% by microscopy) [23] and Malawi (> 37% by RDTs) [24]. However, a lower prevalence was reported among under-five children in Kenya (4.8% by microscopy) and Madagascar (7.8% by RDTs) [25, 26].

Gametocyte carriage by approximately one-third of under-five children with microscopically confirmed *P. falciparum* in the rural communities of Al-Mahweet poses a major challenge to malaria control and elimination efforts. This gametocyte reservoir may considerably contribute to the transmission of infection in the community [5, 6, 27], considering that under-five children have been estimated to comprise 14% of the Yemeni population in 2020 [28]. Because gametocytes were detected microscopically in the present study, the reservoir of sub-microscopic gametocyte carriage can even be larger and is yet to be assessed using molecular surveys. It is important to identify and treat sub-microscopic gametocyte carriers with transmission-blocking drugs to eliminate the disease. Yemen’s NMCP has updated the national antimalarial treatment policy through the integration of low-dose primaquine (PQ) with first- and second-line treatments of uncomplicated falciparum malaria [29]. However, gametocytes among asymptomatic carriers and sub-microscopic gametocyte carriage could compromise this transmission-blocking strategy [30, 31].

The independent prediction of falciparum malaria in children aged three years or older in the present study is consistent with those recently reported for under-five children in Rwanda, Uganda and Malawi [19, 21, 24] but inconsistent with that reported for Nigerian children [23]. In contrast to the present finding, age was not a predictor of falciparum malaria among Yemeni schoolchildren in Bajil [9]. The lower risk of infection with *P. falciparum* in younger children could be partly explained by the greater care they are given, reducing their exposure to mosquito bites. Furthermore, age-related differences in infection of children with *P. falciparum* could be attributed, among other factors, to variations in acquired immunity, transmission intensity and seasonality [32, 33]. The absence of association between the gender of under-five children and falciparum malaria in the present study is consistent with that reported for under-five children in Uganda [21]. Likewise, gender did not predict falciparum malaria among schoolchildren in Bajil [9]. Gender indifferences regarding infection with *P. falciparum* among under-five children in the present study could be explained by similar exposure patterns to mosquito bites among male and female children in rural communities. On the other hand, the non-significant association between the educational and employment status of fathers and mothers with falciparum malaria among under-five children is inconsistent with those reported for children infected with malaria in African countries [26, 34, 35]. However, the lack of association could be attributed to the very large proportion of parents with the same low levels of education and employment type.

The absence of a significant association between fever or other clinical features and falciparum malaria among under-five children in the present study is inconsistent with the finding among schoolchildren in Hajr valley in Hadhramout [36], where fever was significantly associated with malaria. However, the finding of the present study is consistent with the observations among children with malaria in Rwanda, Uganda and Papua New Guinea [19, 37, 38]. The large proportion of asymptomatic cases among under-five children in the present study (28 out of 39 microscopic cases) poses a big challenge to malaria elimination, hindering the strategy of using a single dose of PQ for interrupting transmission.

Vector control through mass distribution of long-lasting insecticidal nets (LLINs) is one of the most important interventions to protect local communities at risk of malaria. In Yemen, LLINs were first introduced and distributed to pregnant women and under-five children in 2006 [14], and since then, this strategy has been increasingly escalated in endemic areas by the NMCP. However, more than half of under-five children in the present study were reported to not have slept under mosquito nets during the night preceding the survey. The low utilization of mosquito nets by targeted communities undermines the effectiveness of LLINs as a prevention and control intervention. In this regard, 42.5% of under-five children with access to LLINs were found to have slept under them in Hodeidah governorate [39]. The reasons for not sleeping under mosquito nets despite having access need to be investigated and addressed to foster the desired impact of LLINs on malaria control and elimination. In accord with the low utilization of mosquito nets, falciparum malaria among children in the present study was found to be independently predicted by not sleeping under mosquito nets. Children who did not sleep under mosquito nets were eightfold more likely to have been infected with *P. falciparum* compared to those who did. Sleeping under mosquito nets was found to be significantly associated with reduced malaria prevalence among under-five children in African countries [19, 23, 24]. In contrast, sleeping under mosquito nets was not significantly associated with a reduction in the prevalence of falciparum malaria among Yemeni schoolchildren in Bajil [9] and under-five children in Uganda [21].

In Yemen, IRS was scaled up as a vector control intervention in 2007 to reinforce efforts to control and eliminate malaria in endemic areas of the country [14]. In the present study, children living in households not sprayed with residual insecticides within the year preceding the survey were fourfold more likely to have been infected with *P. falciparum* compared to those living in sprayed households. The lack of IRS during the year preceding the survey independently predicted falciparum malaria among under-five children in Al-Mahweet rural communities, highlighting the need to assess the household coverage with IRS in targeted areas and take action accordingly. In agreement with the present study, lacking IRS was found to be significantly associated with a higher risk of malaria among Ugandan under-five children [21]. Combining IRS and insecticide-treated nets could be more effective for controlling malaria than either intervention alone [40], and such a combination is recommended in areas of low-to-moderate transmission to reduce seasonal malaria transmission and support elimination efforts [41]. Understanding vector bionomics and transmission dynamics is vital to tailor effective interventions for malaria prevention and control in the rural areas of Al-Mahweet. In another context, an earlier study in Taiz revealed the predominance of endophilic vectors of malaria and recommended the reinforcement of vector control measures that target indoor biting vectors [42].

Sleeping outdoors at night was an independent predictor of infection with *P. falciparum* among under-five children in the present study, with children sleeping outdoors being 4.4-fold more likely to have been infected compared to those sleeping indoors. Given that sleeping outside houses is a common practice in rural communities of malaria-endemic areas of the country because of the hot climate, understanding the exophilic/exophagic behaviours of mosquitoes would help prevent outdoor malaria transmission. Sleeping patterns in the community can affect vector-human contact and could significantly increase malaria transmission. Therefore, parents should be educated not to leave their children to sleep outdoors without using LLINs, particularly with the finding that none of the under-five children in the present study was reported to be protected with mosquito repellents. The absence of a significant association between residence close to water or garbage collections and infection of under-five children with *P. falciparum* in the present study could be attributed to the almost similar geographical and ecological aspects of rural areas. In contrast, residence near water collections was an independent predictor of falciparum malaria among schoolchildren in Bajil [9]. In another context, residents near water collections in Hadhramout governorate, east of Yemen, were at a significantly higher risk of malaria [43].

Given that the strongest risk factors of malaria in under-five children were related to malaria control measures rather than natural topographical or other reasons, there is a need to assess the factors negatively influencing the performance of the NMCP in implementing such interventions. With the great global emphasis on expanding the use of current malaria control interventions together with international funding for them, the strong association between malaria prevalence and poor malaria control measures should raise concerns about the status of malaria control in such rural districts. Accordingly, gaps in coverage, access and utilization of LLINs besides household coverage with IRS should be identified and addressed, considering the potential impact of armed conflicts and the humanitarian crisis on malaria control efforts.

RDTs have become essential tools for malaria control and elimination in rural areas and primary healthcare settings, where microscopy is unavailable or impractical, by enabling immediate diagnosis and prompt treatment. The significant and very good agreement between thick-film microscopy and RDTs for diagnosing falciparum malaria among under-five children in rural communities of Al-Mahweet supports using RDTs in remote and rural areas of the governorate where electricity is unavailable. However, the difference between the results of microscopy and RDTs in six cases justifies the need to assess the sensitivity and specificity of RDTs, preferably compared to PCR. Apart from being time-consuming, the sensitivity of microscopy may overlook infections with low parasite densities and its specificity is affected by the individual capacities of microscopists [44, 45]. Accordingly, it may underestimate the burden of malaria, particularly in asymptomatic low-parasitaemia or sub-microscopic infections. RDTs are useful in augmenting malaria diagnosis by addressing the disadvantages of microscopy, particularly in remote malaria-endemic areas. In the context of the escalating humanitarian crisis and complex emergency, integrated community case management (iCCM) was launched to train healthcare workers in treating under-five children for pneumonia, diarrhoea and uncomplicated malaria in rural and hard-to-reach communities of Yemen in 2017 [46]. However, the success of iCCM as a strategy to control malaria relies on the availability of high-quality RDTs to diagnose malaria in febrile children [47].

The present study is limited by adopting microscopy and RDTs for malaria diagnosis. As a result, overlooking sub-microscopic infections and false positivity of RDTs could not be ruled out. However, it provides preliminary data on the burden of malaria among under-five children in rural communities of Al-Mahweet. Another limitation could be introduced by the possible variations in cluster sizes because of having no information about the population size per cluster to adopt the probability proportional to size sampling approach. As a conservative approach, we used a sample design effect of 2.0 when calculating the sample size to account for the uneven distribution of the outcome in clusters. The findings of the present study can guide public health authorities in tailoring interventions to reduce malaria burden. Malaria prevalence among under-five children in the present study underscores the need for molecular surveillance of malaria among such an at-risk population to uncover the burden of sub-microscopic infections and develop appropriate elimination strategies accordingly.

## Conclusions

Approximately one in ten under-five children in rural communities of Al-Mahweet is infected with *P. falciparum* based on microscopy and RDTs. Age of three years or older, not sleeping under mosquito nets, sleeping outdoors at night and absence of IRS are the independent predictors of falciparum malaria among under-five children. The very good agreement between thick-film microscopy and RDTs for diagnosing falciparum malaria among children supports the usefulness of using RDTs in such resource-limited rural communities.

## Data Availability

Data are available within the manuscript and can be provided by the corresponding author.

## Abbreviations

AOR: Adjusted odds ratio
CI: Confidence interval
COVID-19: Coronavirus diseases 2019
eDEWS: Electronic Disease early warning system
HRP2: Histidine-rich protein 2
iCCM: Integrated community case management
IQR: Interquartile range
IRS: Indoor residual spraying
LLIN: Long-lasting insecticidal net
NMCP: National Malaria control programme
OR: Odds ratio
PCR: Polymerase chain reaction
pLDH: Parasite lactate dehydrogenase
PQ: Primaquine
RDT: Rapid diagnostic test
SPSS: Statistical package for the social sciences
WHO: World Health Organization
